# Native range estimates for red-listed vascular plants

**DOI:** 10.1038/s41597-022-01233-5

**Published:** 2022-03-29

**Authors:** Jan Borgelt, Jorge Sicacha-Parada, Olav Skarpaas, Francesca Verones

**Affiliations:** 1 Industrial Ecology Programme, Department of Energy and Process Engineering, Norwegian University of Science and Technology (NTNU), Trondheim, Norway; 2 Department of Mathematical Sciences, Norwegian University of Science and Technology (NTNU), Trondheim, Norway; 3 Natural History Museum, University of Oslo, Oslo, Norway

**Keywords:** Biodiversity, Biogeography, Ecological modelling, Sustainability, Data publication and archiving

## Abstract

Besides being central for understanding both global biodiversity patterns and associated anthropogenic impacts, species range maps are currently only available for a small subset of global biodiversity. Here, we provide a set of assembled spatial data for terrestrial vascular plants listed at the global IUCN red list. The dataset consists of pre-defined native regions for 47,675 species, density of available native occurrence records for 30,906 species, and standardized, large-scale Maxent predictions for 27,208 species, highlighting environmentally suitable areas within species’ native regions. The data was generated in an automated approach consisting of data scraping and filtering, variable selection, model calibration and model selection. Generated Maxent predictions were validated by comparing a subset to available expert-drawn range maps from IUCN (n = 4,257), as well as by qualitatively inspecting predictions for randomly selected species. We expect this data to serve as a substitute whenever expert-drawn species range maps are not available for conducting large-scale analyses on biodiversity patterns and associated anthropogenic impacts.

## Abstract (summary)

**Table:** 

Measurement(s)	species distributions
Technology Type(s)	machine learning
Sample Characteristic - Organism	Tracheophyta
Sample Characteristic - Environment	terrestrial natural environment

## Background & Summary

Life on Earth is essential to human society as it forms the foundation of present welfare^1^. The growing human population, modern lifestyles and associated pressures on the planet have already resulted in a significant loss of natural habitat and are threatening biodiversity^2–6^. Different initiatives promote the protection of biodiversity and aim to halt its loss, such as the UN Sustainable Development Goals^7^, the Intergovernmental Science-Policy Platform on Biodiversity and Ecosystem Services^8^ and the International Union for the Conservation of Nature (IUCN). Different decision-support tools can contribute to this by assessing environmental performances of products, strategies and policies^2,9–11^. For the development of such tools, but also for the implementation of global conservation strategies and policies itself, spatial data, e.g. in the form of distribution maps of individual species^12^, are crucial. However, besides many species remaining undiscovered or undescribed, we still lack spatial information for most of the ones we know^13^. Consequently, comprehensive and ready-to-use datasets for large-scale analyses are only available for a few vertebrate groups^14–16^. This is concerning, as global conservation strategies and biodiversity impact assessments are limited to these groups, while some hyperdiverse species groups, such as plants, are often not considered^17,18^.

**Fig. 1:**
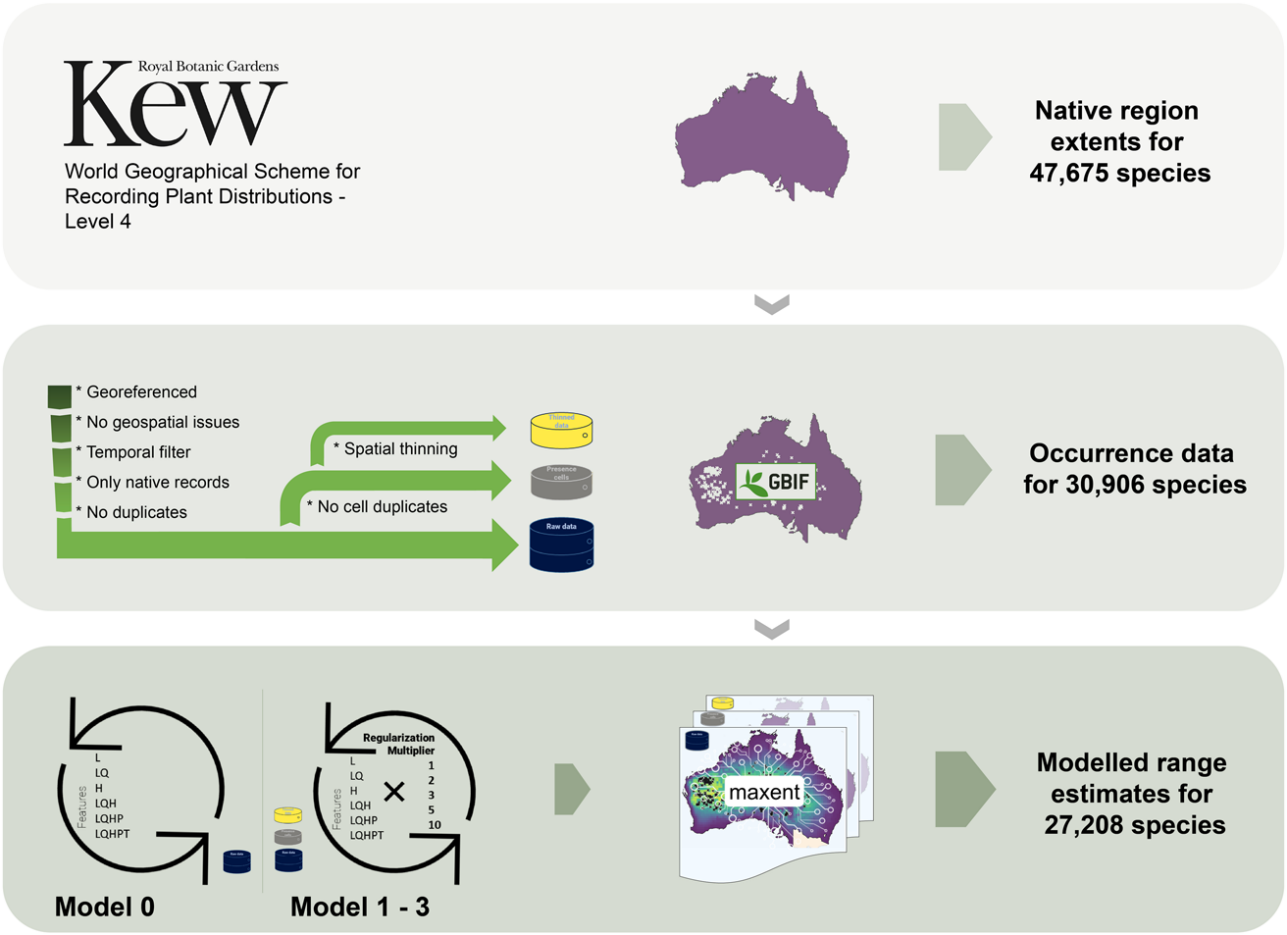
Schematic summary of the dataset. Top: Native region extents were retrieved from Kew’s Plants of the World online. Middle: Occurrence data was retrieved from the Global Biodiversity Information Facility (GBIF)^24^ and filtered into three different occurrence data types: raw data (blue), presence cells (grey) and thinned data (yellow). Bottom: The different occurrence data types were used in Maxent models to predict relative environmental suitability indices within native regions (i.e. range estimates). Differences between Model 0 and Model 1 to 3. Model 0 was trained to support variable selection using raw data in k-fold cross validated Maxent models (one model for each combination of feature classes, i.e. linear (L), quadratic (Q), hinge (H), product (P) and threshold (T)). The selected variables and each of the three occurrence data types were used to train a set of separate k-fold cross validated Maxent models (one model for each possible combination of feature classes, regularization multipliers and occurrence data type). The overall best performing model was selected for each species based on performance metrics.

**Fig. 2:**
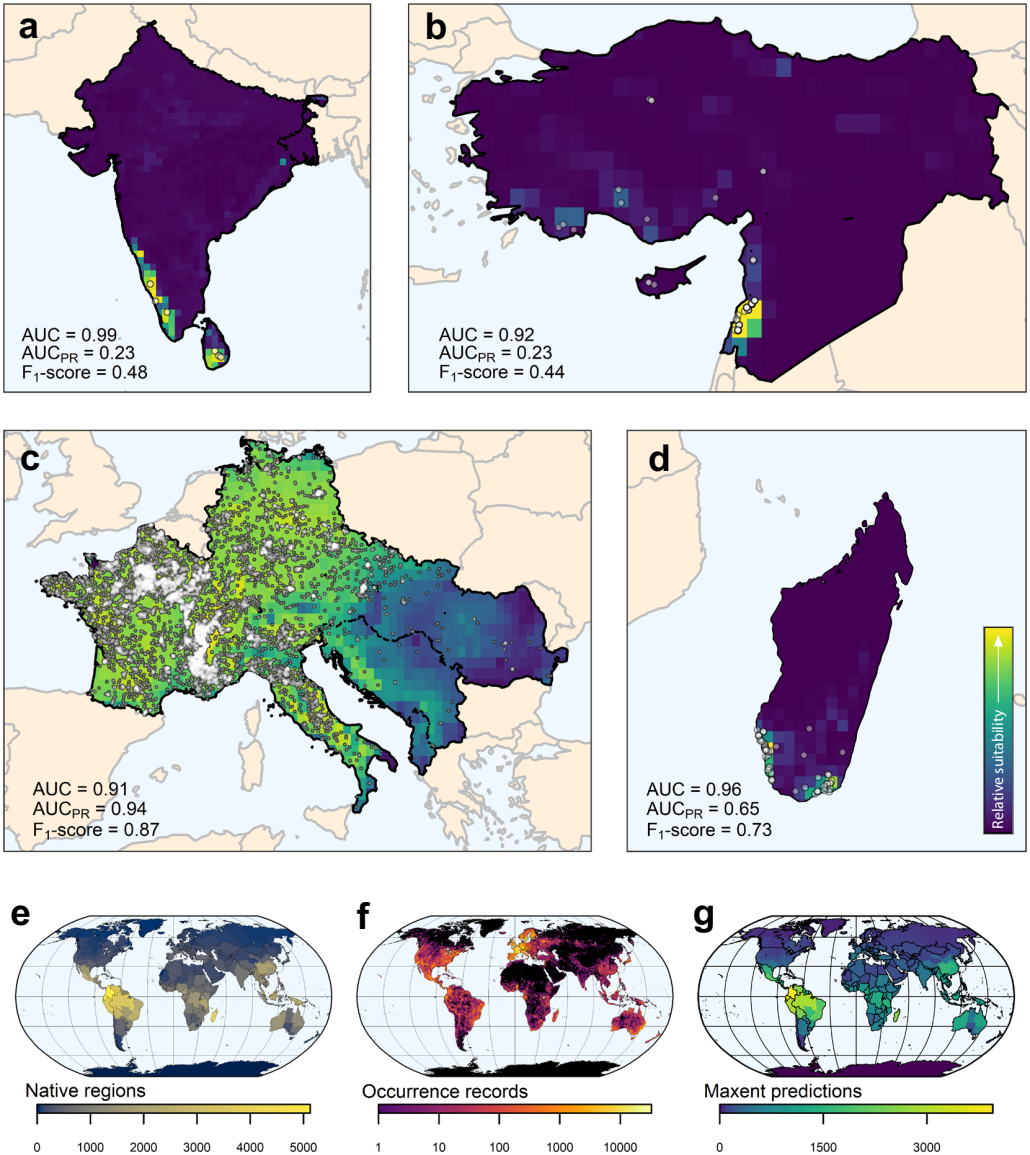
Data examples for randomly selected species and spatial coverage of the dataset. Best performing Maxent prediction, highlighting environmentally suitable conditions within the species native regions (i.e. modelling extent) along retrieved occurrence records (white points) for (**a**) *Amomum pterocarpum*, (**b**) *Cedrus libani*, (**c**) *Laburnum anagyroides*, (**d**) *Megistostegium nodulosum*. Performance of the shown predictions indicated by maximum F_1_-score and the area under the receiver operating characteristics curve for true vs. false positive rate (AUC) and recall vs. precision (AUC_PR_). Bottom: number of (**e**) retrieved native regions, (**f** ) retrieved occurrence records, and (**g**) generated Maxent predictions across the globe.

Here, we provide spatial distribution data for a large fraction of red-listed terrestrial vascular plant species at different levels of spatial detail (Fig. 1), i.e. native regions (n = 47,675), occurrence records (n = 30,906) and modelled range estimates (i.e. a predicted relative environmental suitability^19^ within native regions; n = 27,208). The workflow included data scraping and filtering, as well as variable selection, model calibration and model selection, aiming for best practice^20–22^ but within the constraints of data limitations and computational feasibility at this scale. Species-specific native regions were retrieved from a scheme specifically developed to challenge the lack of distributional knowledge for plant species^23^. Available native occurrence records were retrieved from the Global Biodiversity Information Facility (GBIF)^24^ and subsequently filtered. Range estimates were generated using maximum entropy modelling^19,25–27^, and show where environmentally suitable conditions exist within each species’ native regions (Fig. 2a–d).

The underlying occurrence data is known to be highly spatiotemporally aggregated and variable across administrative borders for some species^28–31^. We aimed at counteracting a potential sampling bias by using three differently treated occurrence data types (i.e. different degree of spatial filtering: no filter, presence cells, thinned presence cells), and by dividing occurrence data in equally-sized bins during model calibration^32^. Up to 96 different models were fitted per species to find optimal variables, model settings and data type. The best prediction was selected for each species based on common performance metrics (i.e. AUC and AUC_PR_).

However, some predictions will undoubtedly remain flawed by underlying biases. Based on comparisons to expert-drawn range maps available from IUCN (n = 4,257) and qualitative inspection of predictions for randomly selected species, we expect this to mainly influence widespread and common species, and hence, only affect the smallest proportion of global biodiversity^33^. In addition, the species most vital for assessing anthropogenic impacts or for defining conservation priorities, are more likely to be small-ranged and endemic. Although validating each prediction was not feasible, we found most individually inspected predictions to either offer an improvement compared to elsewhere available data or an acceptable substitute, although at a coarser spatial resolution and less detailed.

We want to stress that the presented dataset is generated for the purpose of global spatial screening studies and for building a basis for future, global biodiversity impact assessment models. In concert with powerful, species-specific trait and conservation-related databases, the provided data can benefit future work, such as assessing global extinction probabilities^34^, effects of terrestrial acidification^35^, drivers of invasion success^36^, progress towards reaching global conservation goals^37^ and act as pre-assessment prior to expert-based range map generation and red list assessments^38–41^. With a continuously increasing availability of species occurrence records, the presented dataset can be updated frequently to illustrate the state of knowledge at any time. With more data becoming available, precision is likely to increase in the future.

## Methods

### Taxonomic scope

A species list containing all terrestrial vascular plants (n = 52,372) of the global IUCN red list was retrieved from IUCN in April 2021, IUCN version 2021-1^16^. We retrieved each species’ accepted name from Plants of the World Online (POWO)^42^ to facilitate communication to various data portals using the package *taxize*^43^ in R^44^. Plant family, order and class were retrieved from the Integrated Taxonomic Information System^45^ using the package *taxize*^43^ in R. Only species outside the IUCN threat categories “Extinct” and “Extinct in the Wild” were kept, and all species considered as subspecies or varieties according to POWO removed. We attempted to assemble spatial data for each of the remaining 48,144 species.

### Native regions

Species-specific native regions (Fig. 1) were retrieved from POWO using a customized web-scraper function (see section *Code Availability*) and the packages *taxize*^43^ and *rvest*^46^ in R. The data follows the World Geographical Scheme for Recording Plant Distributions (WGSRPD)^23^ and includes a continental, country and regional level. Retrieved WGSRPD-*regions* were matched to its corresponding shapefile at level 4, available from the Biodiversity Information Standards GitHub repository^47^ and rasterized at 30 arc minutes spatial resolution (approximately 56 km at the equator).

### Occurrence records

For species with given native extents in POWO, the maximum number of most recent occurrence points (i.e. 100,000) per native WGSRPD-*country* was retrieved from the GBIF application programming interface (API) using the package *rgbif* ^48^ in R (the equivalent full dataset^49^ is available at 10.15468/dl.uvd56q). The considered environmental variables have changed tremendously in the past decades^50,51^ and only cover a limited period of time, i.e. the years 1979–2013 and 2015 respectively (see section *Environmental data*). Therefore, only records between the years 2000 and 2020 were considered to temporally align occurrence data to both sets of environmental variables as best as possible. If less than 25 records were available for a given species after the year 2000, no temporal filter was set to maximize data retrieval. GBIF records without specified coordinates and with flagged geospatial issues^48^ were not considered. As such, we expect inaccurate coordinate notations as well as records of specimens preserved in museums or other biodiversity facilities to be typically detected. Only points inside reported native WGSRPD-*regions* were kept and duplicated records were removed (hereafter: raw data). The number of raw data records was counted per cell (30 arc min.) using the package *raster*^52^ in R.

### Maxent predictions

We generated spatial predictions within species’ native WGSRPD-*regions* at 30 arc min. resolution (approximately 56 km at the equator) using maximum entropy modelling (Maxent)^19,26,27^, for all species with at least 5 raw data records^53,54^ that were distributed across at least 3 cells, and a native region extent of at least 9 cells. Although an arbitrary threshold, we attempted to allocate computational resources to more meaningful predictions, modelled across larger extents. Maxent is a probability density estimation approach widely used for predicting species distributions based on presence-only data^55^. Background information, required to fit response curves^56^, was collected from each cell within each species’ native regions^57^. For generating models we utilized a high-performance computing infrastructure^58^ allowing for parallel computations using the Maxent software^25^ via R packages *dismo*^59^ and *ENMeval*^60^.

#### Environmental data

We downloaded all CHELSA bioclimatic variables^61,62^ (n = 19, see Table 1 for full list) in 30 arc seconds resolution and aggregated, for computational efficiency, to the chosen modelling resolution (30 arc min.) by averaging. CHELSA bioclimatic variables are a set of modelled, biologically relevant, climatic variables based on data collected during the years 1979–2013^61^. In addition, fractions for different natural land cover types, including different types and mosaics of forest, shrubland, grassland and sparse vegetation, (n = 17, see Table 1 for full list) were calculated based on the European Space Agency’s land cover product for the year 2015 in 300 m resolution^63^. Each land cover class was transformed into a binary raster depicting presence (=1) and absence (=0) of the land cover type. The binary raster was then aggregated to modelling resolution by averaging, resulting in one raster for each land cover class, representing the proportion of land covered by that class per pixel.

**Table 1:** Environmental data used in this study. The layers (n = 36) are based on Karger *et al*.^62^ and the European space agency’s land cover product^63^.

Variable	Code
Annual Mean Temperature	CHELSA_BIO1
Mean Diurnal Range	CHELSA_BIO2
Isothermality	CHELSA_BIO3
Temperature Seasonality	CHELSA_BIO4
Max Temperature of Warmest Month	CHELSA_BIO5
Min Temperature of Coldest Month	CHELSA_BIO6
Temperature Annual Range	CHELSA_BIO7
Mean Temperature of Wettest Quarter	CHELSA_BIO8
Mean Temperature of Driest Quarter	CHELSA_BIO9
Mean Temperature of Warmest Quarter	CHELSA_BIO10
Mean Temperature of Coldest Quarter	CHELSA_BIO11
Annual Precipitation	CHELSA_BIO12
Precipitation of Wettest Month	CHELSA_BIO13
Precipitation of Driest Month	CHELSA_BIO14
Precipitation Seasonality	CHELSA_BIO15
Precipitation of Wettest Quarter	CHELSA_BIO16
Precipitation of Driest Quarter	CHELSA_BIO17
Precipitation of Warmest Quarter	CHELSA_BIO18
Precipitation of Coldest Quarter	CHELSA_BIO19
Fraction of mosaic cropland/natural vegetation	X30_ESA_CCI
Fraction of mosaic natural vegetation/cropland	X40_ESA_CCI
Fraction of broadleaved evergreen, closed to open, tree cover	X50_ESA_CCI
Fraction of broadleaved deciduous, closed to open, tree cover	X60_ESA_CCI
Fraction of needleleaved evergreen, closed to open, tree cover	X70_ESA_CCI
Fraction of needleleaved deciduous, closed to open, tree cover	X80_ESA_CCI
Fraction of mixed leaf type tree cover	X90_ESA_CCI
Fraction of mosaic tree and shrub/herbaceous cover	X100_ESA_CCI
Fraction of mosaic herbaceous cover/tree and shrub	X110_ESA_CCI
Fraction of shrubland	X120_ESA_CCI
Fraction of grassland	X130_ESA_CCI
Fraction of lichens and mosses	X140_ESA_CCI
Fraction of sparse vegetation	X150_ESA_CCI
Fraction of tree cover, flooded, fresh or brakish water	X160_ESA_CCI
Fraction of tree cover, flooded, saline water	X170_ESA_CCI
Fraction of shrub or herbaceous cover, flooded, fresh/saline/brakish water	X180_ESA_CCI
Fraction of bare areas	X200_ESA_CCI

#### Occurrence data types

For some species, several raw data records can be in the same cell at the given spatial resolution (30 arc min.). Although pseudo-replication can inflate model performance (here: during model calibration) and, hence, increases the risk of overfitting, we argue that these occurrence points still contain valid information if they are discrete observations and therefore kept this data. However, we henceforth applied two filters to counteract potential spatial biases, as well as pseudo-replication (Fig. 1). We removed all cell-duplicates from the raw data (hereafter: presence cells), and we applied spatial thinning with a minimum distance of two cells on the presence cells (hereafter: thinned data). Occurrence data was spatially filtered using the R package *spThin*^64^.

#### Model training

A set of Maxent models was fitted for each species using the differently treated occurrence data types. All models were calibrated using k-fold cross validation. The employed occurrence data was partitioned into training and testing bins. For species with only few data points (n < 25), we used k - 1 Jackknife partitioning (k = n)^54^. For species with more data points (n ≥ 25) we used block partitioning (k = 4) to account for spatial autocorrelation of occurrence points in larger datasets^32^. This partitioning splits the occurrence data at a longitudinal and latitudinal line, resulting in approximately equally sized bins^60^.

An initial model (Fig. 1; Model 0) was trained to support the selection of uncorrelated environmental variables using the raw data and all environmental variables (n = 36) for each species. Separate models, one for each possible combination out of all included feature classes (i.e. environmental variables and transformations thereof), were trained. We included linear (l), quadratic (q), product (p), hinge (h) and threshold (t) transformations, resulting in 6 possible combinations (i.e. l, lq, h, lqh, lqhp, and lqhpt). The best performing model was selected based on the corrected Akaike information criterion (AICc)^65–67^. However, if no model performed best in terms of AICc, or if this metric was unavailable for 50% of fitted models, the average testing area under the receiver operating characteristics curve (AUC; see section *Technical Validation*) during model calibration was used instead. Permutation importance was retrieved for all variables in Model 0. Correlated variables were identified using Spearman’s rank correlation coefficient (ρ) and defined as ρ ≥ | ± 0.7|. In any set of correlated variables, only the variable with the greatest permutation importance was kept.

The selected environmental variables were used to train separate models for each of the three differently treated occurrence data types: raw data (Model 1), presence cells (Model 2), and thinned data (Model 3). Model 1 was trained if at least 5 raw data records were available, distributed across at least 3 cells (see above). Model 2 and Model 3 were trained if at least 3 records of the corresponding data type were available to avoid computational failure. Although a smaller sample size, we argue that if those models performed better than Model 1, the threshold of 5 records becomes arbitrary and the assessed performance indicators (see section *Technical Validation*) more valuable. The same model architecture as in Model 0 was utilized, including model calibration and selection of the best performing model. However, this time, we added five different regularization multipliers (RM; i.e. 1, 2, 3, 5 and 10; based on previous studies^68–70^) to counteract overfitting^20,56^ and for building simpler, ecologically more relevant, models^60^. Hence, separate models for each possible combination out of feature classes and RMs were trained (Fig. 1; Model 1–3), resulting in 30 trained models for each data type and up to 90 models per species.

### Metadata

Metadata was assembled for all data and includes general information about species (taxonomy and red list status), provided data type (native regions, occurrence records or Maxent prediction), bounding box of native regions, and if relevant, information about the occurrence data (number of raw data records, Moran’s Index^71^, calculated as a measure of spatial autocorrelation and based on the number of raw occurrence points obtained per cell), and Maxent metadata: training data (filter treatment, number of training data points), thresholds for converting the prediction into binary range maps^59^, model settings (features, parameters, transformations, regularization multiplier, variables) and out of the box^60^ model performance, including degree of overfit (DOO) quantified as the difference between calibration and testing AUC during k-fold cross validation^70^, as well as self-assessed model performance metrics as described in the section *Technical Validation*.

## Data Records

### Dataset

The presented dataset is stored in a stable Dryad Digital Repository^72^ and can be explored at https://plant-ranges.indecol.no. The dataset includes spatial information for 47,675 species at different levels of detail. In total, range estimates (i.e. relative environmental suitability within native regions) have been predicted for 27,208 species using Maxent, for 30,906 species native occurrence records are provided, and for 47,675 species the spatial extent of its native WGSRPD-*regions* is provided.

All gathered and generated data are stored in netCDF files and can be called by specifying a *varname*. Spatial predictions are provided in Maxent’s raw as well as default output (i.e. complementary log-log (cloglog) transformed, but see section *Usage Notes*)^27,59,60^. The suggested data is stored in folder *basic*. These netCDF files (default output and raw output) assemble the best performing Maxent prediction (*varname*: Maxent prediction) for each species selected based on the highest harmonic mean between AUC and AUC_PR_ (see *Technical Validation*), along with number of occurrence records per cell (*varname*: Presence cells) and rasterized native WGSRPD-*regions* (*varname*: Native region).

The netCDF files in folder *advanced* contain one Maxent prediction for each occurrence data type (*varname*: Model 1, Model 2 or Model 3), instead of best performing Maxent prediction (i.e. *varname* Maxent prediction is not applicable). Number of occurrence records per cell (*varname*: Presence cells) and rasterized native WGSRPD-*regions* (*varname*: Native region) are identical in all netCDF files.

Each band in the netCDF files assembles the mentioned variables for one species. The corresponding bands can be looked up in the metadata (i.e. *speciesID*). Furthermore, the metadata can be used to select appropriate cut-off thresholds for generating binary range maps, filter models based on species, performance, or desired datatypes, and to lookup the relevant study extent for masking individual predictions (see *Usage Notes*).

## Technical Validation

### Maxent predictions

We calculated performance metrics for model 1 to 3 for each species using its corresponding presence cells to validate the Maxent predictions. Receiver operating characteristic curves and the corresponding area under the curve for *recall* (i.e. *true positive rate, sensitivity*) versus *false positive rate* (AUC) as well as *precision* versus *recall* (AUC_PR_) were generated using the packages *ROCR*^73^ and *PRROC*^74^ in R. *Recall* was calculated as the fraction of correctly predicted presence cells compared to all presence cells of the reference (Eq. 1), the *false positive rate* as the fraction of falsely assigned presence cells compared to all true absence cells (Eq. 2), and *precision* as the fraction of correctly assigned presence cells compared to all predicted presence cells (Eq. 3). In addition, F_1_-scores (Eq. 4) were calculated as harmonic mean between *recall* and *precision* at all possible cut-off thresholds to transform the Maxent prediction into a binary range map. The maximum obtained F_1_-score indicates how well a potential binary range map performs at equal importance of *recall* and *precision*.

1 $$Recall=\frac{True\;Presence}{True\;Presence+False\;Absence}$$

2 $$False\,positive\,rate=\frac{False\,Presence}{False\,Presence+True\,Absence}$$

3 $$Precision=\frac{True\;Presence}{True\;Presence+False\;Presence}$$

4 $${F}_{1}=2\left(\frac{precision\cdot recall}{precision+recall}\right)$$

AUC and AUC_PR_ are threshold-independent performance measures for binary classifiers. An AUC value of 1 indicates a perfect model, an acceptable AUC value (>0.7)^75^ indicates the ability to predict many true presences at a low false positive rate, and an AUC value of 0.5 indicates the model performing as good as a random guess. The average AUC obtained across the suggested dataset was 0.95 when comparing predictions to its corresponding presence cells (Table 2), indicating well-performing models for the majority of species. For 26,977 species (99%), at least one Maxent prediction had an AUC value above 0.7^75^.

**Table 2:** Performance of Maxent predictions in the suggested dataset. Mean and median values of area under the receiver operating characteristics curve for true vs. false positive rate (AUC) and recall vs. precision (AUC_PR_) for all species and across different IUCN threat categories (i.e. data-deficient (DD), least concern (LC), near-threatened (NT), vulnerable (VU), endangered (EN) and critically endangered (CR)). Calculations are based on presence-background data (n = 27,208) and on comparison to expert-based range maps retrieved from IUCN (i.e. reference range, n = 4,257).

	Reference		Red list category						
			DD	LC	NT	VU	EN	CR	Total
AUC	Presence - background	Mean	0.939	0.937	0.95	0.96	0.971	0.957	0.945
		Median	0.961	0.951	0.977	0.985	0.994	0.989	0.964
	Reference range	Mean	0.817	0.89	0.927	0.931	0.929	0.915	0.902
		Median	0.852	0.925	0.972	0.974	0.98	0.987	0.943
AUC_PR_	Presence - background	Mean	0.576	0.529	0.656	0.69	0.749	0.7	0.589
		Median	0.603	0.535	0.717	0.755	0.833	0.797	0.617
	Reference range	Mean	0.516	0.664	0.686	0.653	0.655	0.592	0.658
		Median	0.527	0.702	0.737	0.712	0.699	0.626	0.702

AUC_PR_ is not affected by true negatives (i.e. true absence) which often dominated our dataset. A higher AUC_PR_ value indicates a relatively higher ability to correctly predict a high proportion of presumably true range while maintaining a high precision compared to a lower AUC_PR_. However, the AUC and AUC_PR_ values, as well as max. F_1_-score, described here were calculated based on presence-background data and are highly influenced by class balances. Strictly speaking, both false presences and true absences cannot be determined with presence-only data. Hence, the performance metrics described here can only be used to compare different models for a given species, but not across different species^76,77^.

**Fig. 3:**
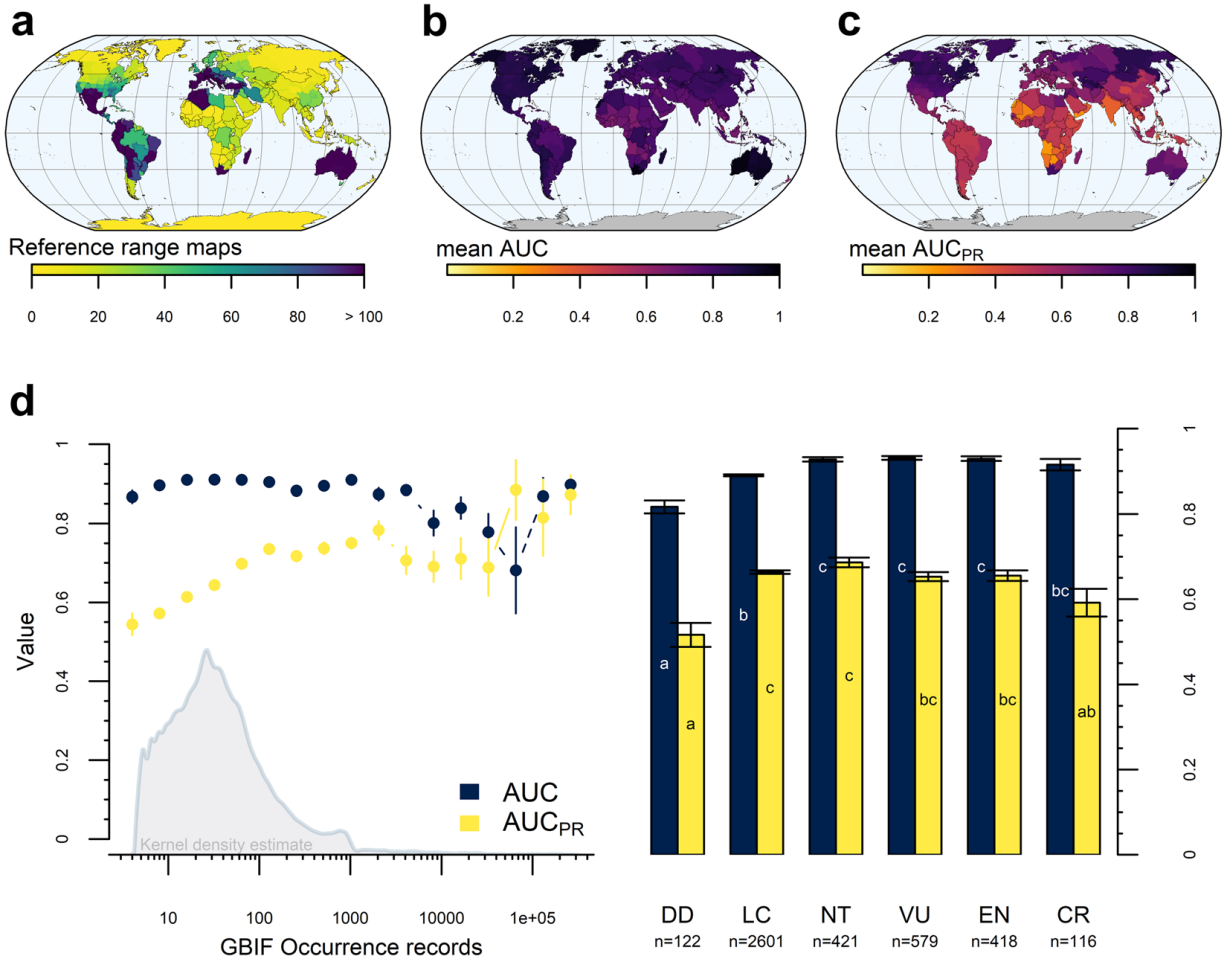
Performance metrics for the suggested Maxent predictions. (**a**) Number of reference range maps available used for calculating performance metrics. Average values for species native to the corresponding regions of area under the receiver operating characteristics curve for (**b**) true vs. false positive rate (AUC) and (**c**) recall vs. precision (AUC_PR_). (**d**) Mean and standard deviation of AUC (blue) and AUC_PR_ (yellow) per rounded log-transformed number of raw occurrence data points (left) and for species in different IUCN red list categories (right), i.e. data-deficient (DD), least concern (LC), near-threatened (NT), vulnerable (VU), endangered (EN) and critically endangered (CR). Significant differences across IUCN categories in d are indicated by different letters in bars for AUC (white text) and AUC_PR_ (black text).

Therefore, we evaluated the Maxent predictions by comparison to available expert-based range maps, as an additional evaluation dataset^32^. Expert-based range maps were retrieved from IUCN, if available (hereafter: reference ranges). Only reference ranges that were labelled as “native” and “extant (resident)” or “probably extant (resident)” were considered. For 4,257 species of our Maxent predictions, range maps were available at IUCN. These species were unevenly distributed in space (Fig. 3a), across IUCN red list categories (Fig. 3d) as well as the plant classes dicots (Magnoliopsida, n = 3,480), monocots (Liliopsida, n = 731), ferns (Polypodiopsida, n = 27), conifers (Pinopsida, n = 17), and lycopods (Lycopodiopsida, n = 2). Reference ranges were used to calculate the above described performance measures (i.e. max. F_1_-score, AUC and AUC_PR_). However, this time we dealt, presumably, with actual presences and absences of the given species, making the performance metrics comparable across species^76^. Maxent predictions for species classified as “data-deficient” (DD) obtained the lowest, and predictions for species classified as “near-threatened” (NT), “vulnerable” (VU) and “endangered” (EN) the highest AUC values (Fig. 3d). However, these differences were marginal and all average values consistently high across different IUCN categories (mean AUC: 0.9; Table 2) and across the globe (Fig. 3b). Although AUC is a strong indication of model performance^75^, the predictions seem to rarely accommodate both a high *recall* and a high *precision* (represented in either max. F1-score or AUC_PR_ value) when compared to reference ranges. However, we found a large variation and no clear trend in AUC_PR_ values for species across different threat-level categories (Fig. 3d), and although the average AUC_PR_ was lowest for species native to parts of central Africa, India and south-eastern Asia (Fig. 3c), we expect these values to be of little explanatory power due to the limited sample sizes in these regions (Fig. 3a). Moreover, AUC_PR_ seems to increase with increasing data availability (Fig. 3d). We assume that low data coverage in sparsely populated areas influenced modelling performance for some, primarily widespread, species, highlighting that sometimes more spatially distributed occurrence data is required for making expert-alike range maps^78^.

Furthermore, based on a qualitative assessment of predictions for twelve randomly selected species, we expect uncertainties due to differences in data availability across administrative borders as well as for highly naturalized species. For instance, the clustered occurrence records for *Cedrus libani* in Lebanon (Fig. 2b) resulted in less precise data than elsewhere available for this species^79^, while the prediction for *Laburnum anagyroides* (Fig. 2c) was affected by naturalized occurrence records outside its native origin^80^ but still within its native WGSRPD-*regions*. However, this will be most problematic for abundant, widespread, and naturalized species, and hence only relevant for the smallest fraction of global biodiversity^33^. In addition, the predictions for more vulnerable species, presumably small-ranged or endemic, seem to perform better than species in the lowest red list category (i.e. least concern (LC)) in terms of AUC when compared to reference ranges (Fig. 3d).

In fact, the remaining randomly selected predictions were either consistent with point data (e.g. *Terminalia macrostachya*^81^), reflected the current knowledge of elsewhere available data, although at a coarser spatial resolution and less detailed (e.g. *Mammillaria grahamii*^82^), or offered an improvement compared to previously unavailable spatial data (e.g. *Eucalyptus elliptica*^83^, *Megistostegium nodulosum*^84^ (Fig. 2d), *Memecylon elegantulum*^85^, *Psidium salutare*^86,87^, *Siparuna conica*^88,89^, *Trisetaria dufourei*^90^). However, the prediction of *Pyracantha angustifolia* was difficult to evaluate due to poorly understood range dynamics^91^, highlighting the need for more data for vascular plant species.

We want to stress that our predictions indicate environmentally suitable conditions even if isolated from known species occurrence locations. For instance, *Amomum pterocarpum* seems to be restricted to southern India and Sri Lanka^92,93^ while our prediction indicates environmentally suitable conditions in north-eastern India (Fig. 2a), which in fact, supports a possible observation nearby^94^. We further detected several expert-based range maps with a substantial mismatch to our data, confirming that some of the expert-based data may be too conservative^95^ (e.g. *Magnolia pugana*)^96^. However, we also found expert-based ranges being smaller (e.g. *Vallesia glabra* or *Tetraclinis articulata*)^97,98^ than predicted environmental suitability indicates, or being incorrectly georeferenced (e.g. *Corylus cornuta*)^99^. Hence, besides highlighting mismatches to expert-based range maps, we expect this dataset to be of sufficient quality to serve as time- and cost-efficient range map substitutes and pre-assessed range estimates for currently unmapped species.

### External data

The retrieved native WGSRPD-*regions* are provided by POWO under a CC BY 3.0 license (https://creativecommons.org/licenses/by/3.0/) and have been checked for consistency to assure proper workflow of data retrieval from POWO and feature matching to the WGSRPD level 4 shapefile. However, the data provider, POWO, cannot warrant the quality or accuracy of the WGSRPD data^42^. In addition, other data (e.g. ecoregions^100^) may ecologically be more relevant than administrative boundaries. However, WGSRPD offers the most detailed data on species’ native origins available on a large-scale, to the best of our knowledge. An attempt in matching native WGSRPD*-regions* to ecoregions was discontinued after loss of information due to incompatible geographical boundaries. Hence, we consider the utilized WGSRPD*-regions*, currently, as the best compromise between level of detail and availability of data on species’ native origins. Furthermore, spatial inaccuracies and biases in the occurrence data retrieved from GBIF were counteracted by the implemented filtering steps, the coarse spatial resolution, by avoiding non-native occurrence records and the model calibration techniques. However, any unforeseen misclassified or misreported records may flaw predictions for individual species. In addition, data retrieval via GBIF’s API was limited to 100,000 occurrence records per request. We extended this limit by sending one request per native country for each species, and hence, expect this issue to be irrelevant for our study. We further want to stress that most of the generated predictions have not been validated individually, and that some predictions may be erroneous either due to data limitations or simply because digitally stored data can contain minor but crucial blunders. For instance, in terms of nomenclature, the red-listed species *Cotoneaster cambricus* is endemic to Wales^101^, but also seems to be a synonym for a widespread species according to POWO^42^. Consequently, either our spatial prediction or the expert-based range for this species is incorrect.

## Usage Notes

All data handling, modelling and visualization was done using R version 4.0.3^44^ in RStudio version 1.4.1103^102^. Handling of all spatial data was done using the R packages *raster*, *rgdal*, *maptools*, *rgeos* and *sp*^52,103–106^. A showcase for opening the different data types for individual species, is available at https://github.com/jannebor/plant_range_estimates. Although functionality of the code may be given at newer, or older, versions, we expect the best user-experience using the versions specified in this descriptor.

Maxent predictions are given as raw and cloglog transformed output. These outputs are related monotonically, meaning that the performance metrics described in this study, as well as a potential binary range map (excluding prevalence dependent thresholds), will be identical for both raw and cloglog output^56^. For users mostly interested in qualitative analyses, both predictions can simply be interpreted as indices of environmental suitability^20^. However, due to rescaling, the exact interpretation and appearance of each output differs. In general, Maxent’s output interpretation depends on the underlying data, and differs, in our case between Model 1 (raw data including pseudo-replicates = abundance) compared to Model 2 and 3 (presence), but gives an estimate of the abundance, or presence, of the species in relation to the true modelled quantity (either abundance or presence). Maxent’s raw output reflects the exponential Maxent model itself, and can be interpreted as a relative occurrence (or presence) rate summing up to 1^20^. The raw output does not rely on any assumptions^20^, however, it may not perform well in visualizing actual differences in suitability^107^. Being rescaled on a more common range from 0 to 1, the cloglog transformation compresses extreme values, and hence facilities visualization and comparison amongst predictions^27^. It can, arguably, be interpreted as a relative probability of presence under certain assumptions^27^. However, as these assumptions are rarely met, we strongly discourage users from this interpretation and suggest interpreting the cloglog output values as an estimate of relative environmental suitability^20^ instead.

We further suggest using Maxent predictions with an AUC below 0.7 only in exceptions, and in large-scale studies. In general, our predictions may overestimate true range extents of endemic species and underestimate ranges of widespread species. However, in worst case, the entire native WGSPRD-*regions* are outlined as being environmentally suitable, which may be acceptable in some cases, but not in others.

In addition, Model 1 has been fitted with the suggested minimum number of records for generating meaningful distributions models^53,54^, but Model 2 and 3 were in some cases trained with less records. Whether this low sample size as well as its implied uncertainty is acceptable or not will differ between users and applications and needs to be considered.

The full data, including Maxent predictions (cloglog transformed), underlying occurrence records, native regions and corresponding metadata, can be explored at https://plant-ranges.indecol.no. Here, the predictions based on individual models (Model 1 to 3) as well as a suggested (i.e. best performing) prediction highlight environmentally suitable conditions, if available for the selected species. Predictions can potentially be transformed into a map indicating where the species is most certainly found, as required for local management and conservation actions^95^, or into a conservative range map, best suited for analysing global patterns^108^ and highlighting where a species is certainly absent^109^. However, the choice of an appropriate cut-off threshold is highly application specific. We outlined “potential range maps” in the data explorer for illustrational purposes only and based on the best performing prediction. We applied different cut-off thresholds to represent different levels of confidence using the R package *dismo*^59^. The threshold at which there was no omission (possibly suitable), the threshold at which the F_1_-score is highest (probably suitable) and presence cells (presence).

## Data Availability

All data and code is available without restrictions under the terms of a Creative Commons Zero (CC0) waiver (https://creativecommons.org/share-your-work/public-domain/cc0/). R code for retrieving and filtering data from POWO and GBIF, and for generating and evaluating Maxent models is available on GitHub (https://github.com/jannebor/plant_range_estimates). Any further requests can be directed to the corresponding author.
